# Entropy-Driven Environmental Impact Assessment of Condensate-Induced Irreversibility in Integrated Building Energy Systems

**DOI:** 10.3390/e28030305

**Published:** 2026-03-09

**Authors:** Mehmet Ziya Söğüt, Zafer Utlu

**Affiliations:** 1 Maritime Faculty, Piri Reis University, İstanbul 34940, Türkiye; mzsogut@pirireis.edu.tr; 2 Department of Industrial Engineering, Faculty of Engineering and Natural Sciences, İstanbul Atlas University, İstanbul 34403, Türkiye

**Keywords:** integrated buildings, entropy, irreversibility, exergy, environmental impact, sustainability

## Abstract

In multifunctional and high-energy-density integrated buildings, energy performance and environmental impacts are affected by the environmental conditions in which they are located. Entropy production, which is an output of exergy analysis in energy performance, offers a new evaluation area for energy management in this context. In the study developed for this purpose, the condensate line formed in the steam distribution lines of an integrated building was modeled, and the possible inefficiency potential of the condensate load formed and the usability of the approach developed over entropy production were suggested by energy management. Entropy production due to exergy destruction of distribution lines derived from condensate pump data in the integrated building was evaluated with two environmental indices developed. According to the analysis, the average exergy efficiency for the distribution lines of the integrated building system is 22%, with exergy extinction reaching 78%, indicating a high level of return level. The recovery potential associated with the total exergy flow was calculated as 50.8%, while the entropy generation potential due to the condensation load was 65.3%. From an environmental perspective, the potential for pollution based on entropy has reached 64.9%, while the target energy efficiency level associated with condensate management has been set at 33.5%. The findings suggest that this approach for energy management offers a quantitative evaluation opportunity between thermodynamic irreversibility and environmental performance in buildings. At the end of the study, a comparative analysis of this approach with the classical regression approach for energy management is also given.

## 1. Introduction

Energy efficiency and energy management have been developed as the main strategic objectives of global decarbonization policies based on the environmental sustainability of energy systems. The increasing energy intensity associated with energy use in buildings reflects not only rising demand but also the environmental pollution dimension of fossil-based energy consumption [1]. As a sector accounting for nearly 40% of total energy consumption worldwide, buildings have been the focus of extensive research aimed at reducing fossil fuel dependency. However, despite these efforts, effective and sufficient solutions have not yet been fully realized, indicating persistent challenges in energy efficiency and energy conservation within decarbonization processes [2]. These challenges largely stem from limited transformation of existing building technologies, particularly regarding thermal inefficiencies and operational shortcomings that continue to constrain overall system performance.

In recent years, the concept of integrated buildings has emerged as a transformative approach encompassing technological, operational, and sustainability-oriented developments throughout the building life cycle, from construction to management [3]. Integrated buildings are characterized by the combination of multiple functions and technologies within a unified operational framework, enabling improved efficiency in multipurpose facilities [4]. With the advancement of building technologies, optimizing the energy systems employed in integrated buildings has become increasingly important for ensuring sustainable operation [5,6]. Nevertheless, uncontrolled feedback mechanisms and inefficiencies in energy distribution systems often result in substantial performance losses, particularly in demand-driven building energy systems. Such inefficiencies cause fluctuations in energy consumption and reduce overall system efficiency. Heating systems, in particular, represent one of the most energy-intensive components of integrated buildings and pose major challenges in terms of environmental impact and economic cost [7,8].

Heating demand in integrated buildings is commonly supplied through central or district heating systems, where steam-based energy distribution remains a widely adopted and conventional solution. Large-scale facilities such as hospitals, campuses, military bases, and educational buildings typically rely on underground steam distribution networks to meet their thermal energy requirements [9]. The efficiency of these distribution networks plays a decisive role in overall energy performance. Heat losses occurring along distribution lines directly reduce system efficiency and increase fuel consumption, thereby increasing emission loads. In addition, minimizing heat losses associated with condensate formation in steam distribution lines of such structures is crucial for achieving emission reduction targets in sustainable energy management. Designing and operating these systems in accordance with environmental management and energy saving objectives is therefore critical for both cost reduction and environmental protection [10,11]. In this context, energy efficiency practices in integrated building management should be regarded as a driving force for decarbonization processes, supported by sustainability-oriented energy management strategies [12,13].

Energy consumption in buildings is closely linked to environmental sustainability goals due to its strong association with greenhouse gas emissions. Fossil fuel use for heating, cooling, and electricity generation leads to significant emissions of carbon dioxide (CO_2_), which is widely recognized as a primary contributor to climate change. Accordingly, improving energy efficiency and integrating renewable energy sources have become key strategies for mitigating the environmental footprint of buildings. The literature reports numerous effective solutions, including innovative structural designs, energy efficient systems, and sustainable materials, aimed at optimizing energy use. However, most of these approaches focus predominantly on reducing the quantity of energy consumption, while offering limited insight into the thermodynamic quality of energy use and the role of irreversible losses within energy distribution systems.

From a broader environmental perspective, emissions originating from fossil fuel use in buildings constitute a critical component of global climate change. Literature studies emphasize the environmental and health risks associated with energy conversion processes and the dominance of non-renewable energy resources, particularly in densely populated urban environments [14]. Integrated building strategies have also been examined in relation to economic development and urban sustainability, highlighting their potential to improve living standards and support regional growth [15]. More recent reviews on smart building energy management underline the importance of advanced optimization techniques while identifying research gaps related to demand-side management and system-level performance assessment [16]. These findings indicate that reducing fossil fuel use in buildings is not only an environmental necessity but also a critical factor in enhancing socio-economic sustainability.

In this context, entropy-based approaches are gaining increasing attention as a robust theoretical framework for assessing energy efficiency and environmental performance in complex energy systems. Entropy is related to the exergy annihilation that occurs as a result of irreversibility and is a value that develops depending on the environmental conditions in which the system is located. Unlike traditional energy-based indicators, the entropy approach is based on the correlation of exergy destruction revealed by exergy analysis with the general exergy input, giving a unitless result of irreversible losses in the system. Heat transfer, which significantly affects system performance in building energy systems, is closely related to heat transfer processes such as fluid flow, as well as heat loss caused by a finite temperature difference, which defines the entropy generation mechanism, such as condensation. When combined with exergy analysis, entropy-based assessment allows for the evaluation of the impact of energy losses as a quantitative value in interaction with the environment. Previous studies have shown that the use of exergy serves as a unifying concept linking environmental impact and the principle of sustainability [17], while entropy generation has been shown to be a quantitative indicator of irreversibility and environmental burden [18,19].

Recent studies published in *Entropy* further emphasize that entropy generation should not be interpreted merely as a thermodynamic loss but also as a quantitative proxy for environmental impact and sustainability performance [20,21]. In this perspective, minimizing entropy generation emerges not only as an efficiency-oriented objective but also as a design and operational criterion for sustainable energy systems. Moreover, entropy-based environmental assessment frameworks have been successfully applied to various energy systems, demonstrating their potential to translate irreversibility into interpretable performance indicators [22].

Despite the growing application of exergy analysis in building energy studies, the clear link between entropy formation, condensate formation in steam distribution lines, and environmental impact has not been adequately explored. Current research shows that energy losses are largely quantitative in relation to energy efficiency, while exergy consumption has the potential to be irreversible due to exergy breakdown, which is affected by dead state conditions. In this process, energy management mostly makes an evaluation based on regression analysis for process efficiencies. But the real question is whether possible losses can be developed as a metric associated with exergy destruction. Can a traceable and evaluable model be proposed for energy management? To address this gap, the present study presented an approach for the holistic assessment of irreversibility in the main steam distribution lines of an integrated building system. A unitless number associated with the generation of entropy due to exergy destruction found by exergy analysis was produced. A model developed based on this approach has contributed to the monitoring criteria of energy management. A data distribution was derived based on the pumping data of the mechanical structure process referenced in the study. A theoretical framework based on exergy and entropy analysis has been developed to assess condensate loads and their contribution to energy inefficiency and environmental impact through an integrated approach. By introducing entropy-based environmental performance indices, this study aims to establish a quantitative bridge between thermodynamic irreversibility and sustainable energy management, providing an approach for energy management in integrated buildings.

## 2. Integrated Buildings and Environmental Sustainability

Integrated structures are increasingly preferred to increase the environmental sustainability of building projects and optimize energy efficiency. The most obvious advantage of this design approach is that it enables all participants in the project to work in a coordinated manner from the inception as a whole, from architecture to engineering, from energy experts to building material suppliers. Unlike traditional design methods, the integrated building design (IBD) process requires multidisciplinary collaboration, making the project process faster, more efficient, and sustainable. Yılmaz [23] emphasizes that this type of design system is an effective method to prevent negatives such as errors, cost increases, and time extensions frequently encountered in traditional approaches. Involving all stakeholders in the decision-making process in the correct order at the beginning of the project allows for more efficient management of the system’s environmental impacts. Thus, the design process proceeds in accordance with environmental sustainability goals at every stage.

The integrated design process and the relationship between the participants are much more complex and interactive than traditional design systems. This process requires intertwined and multifaceted collaboration among participants. In traditional design systems, the design phases are often linear and unidirectional, which increases errors and productivity losses in projects. In integrated design, since all participants are involved in every stage of the process, optimal results are achieved not only in terms of aesthetics and functionality but also in terms of environmental sustainability. In particular, building information modeling (BIM) technology plays an important role in the successful implementation of this process. BIM integrates all processes, from project design to construction, in a digital environment, thus enabling energy efficiency, optimization of material use, and lifecycle analysis of buildings. The use of BIM enables more effective and accurate sustainable building analyses. Additionally, thanks to BIM technology, all project stakeholders can share data in real time, so that problems encountered at any stage of the project are quickly identified and resolved. This makes it easier to achieve the environmental and economic sustainability goals of the project. In such approaches, the entropy generation approach based on exergy destruction supports data management in its analytical aspect of energy management and improves environmental impact potentials in case of instant climate change.

One of the main reasons why the integrated design process has a positive impact on environmental sustainability in buildings is the optimization of systems and building components. Many literature studies reveal that practices such as integrated energy management and environmental impact assessments are more effectively involved in this process. In building design, factors such as the combination of passive and active energy systems, efficient management of water and energy, and conservation of natural resources are among the characteristics of the integrated design process. Innovative approaches used in such projects incorporate environmental sustainability criteria such as energy efficiency, water conservation, and low carbon emissions into the design process from the outset. In this context, integrated design approaches are shaped to consider the entire environmental impact, not just indoor energy systems. The relationship between integrated construction and environmental sustainability establishes a strong connection that enhances efficiency and environmental performance in buildings. Design models in which participants are involved in the early stages and with which they actively interact throughout the process make it easier to achieve environmental sustainability goals. The literature supports that such design processes are effective in reducing the environmental impact of buildings and increase cost efficiency by shortening the completion time of the project. Thus, integrated structures not only save energy but also integrate environmental sustainability into the entire project process, resulting in greater efficiency with less resource usage.

### 2.1. Emission Sources and Inventory Framework in Buildings

Buildings are a major source of energy-related greenhouse gas (GHG) emissions on a global scale, mainly from fossil fuels. As a matter of fact, modeling studies come to the fore for this in all organizations. For example, according to 2023 data, they account for 33% of the EU’s total energy-related emissions. These emissions come from two main sources: first, the direct use of fossil fuels in buildings (e.g., gas and oil used for heating); the second is the indirect effect of fossil fuels on electricity and heat production used in buildings (e.g., energy consumption of electrical appliances, lighting, and cooling systems). For such organizations, accurate monitoring and reporting of emissions play a critical role in understanding and reducing the environmental impact of buildings. The European Green Deal and Renewal Wave Strategy aim to take concrete steps such as reducing emissions from buildings. To achieve these goals, the Energy Performance Buildings Directive (EPBD), revised in 2024, proposes improvements in areas such as the energy efficiency of EU buildings, the decarbonization of heating systems, and the deployment of solar energy systems. In addition, with a new carbon trading system called ETS2, direct emissions from the use of fossil fuels in buildings will be priced from 2027. This will create an incentive mechanism for buildings to use renewable energy and increase energy efficiency, thus reducing carbon emissions. This framework enables EU countries to create a legal basis for reducing emissions in buildings.

**Figure 1:**
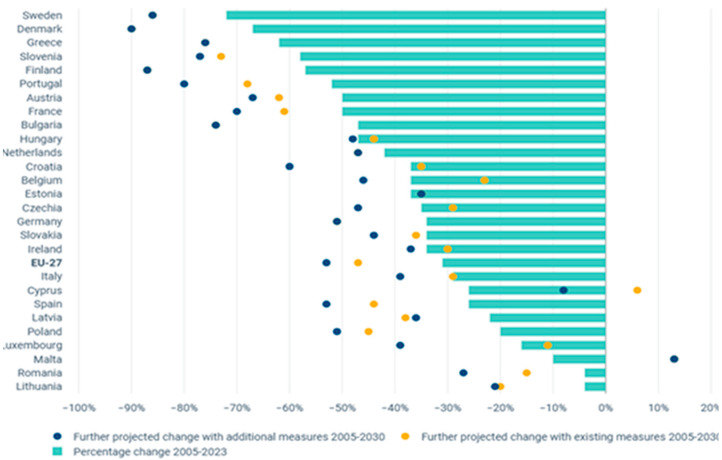
Emission change in EU buildings with global impact [24,25].

Monitoring and reporting emissions in buildings is of great importance for continuous improvements and measuring the effectiveness of policies. As a matter of fact, the effective measures put forward by the EU2 recorded a total emission reduction of 43% for the buildings sector between 2005 and 2023. This reduction has emerged as a result of strategies to improve the energy efficiency of buildings. However, factors such as the growing number of households, increased living spaces, and the use of more electrical appliances have partially offset emissions by increasing energy consumption in buildings. Additionally, cooling needs increase energy consumption due to increased heat waves during the summer. As can be seen in Figure 1, forecasts for 2024 indicate a further slight decrease in direct emissions from fossil fuel use in buildings compared to 2023, in the context of ongoing energy efficiency and decarbonization efforts in heating systems. Member States anticipate that the downward trend in emissions from buildings will continue in the future.

To ensure the environmental sustainability of buildings, the creation of a sensible applicable matrix within the scope of energy management is an important step in systematically evaluating strategic approaches to improve energy efficiency and reduce carbon footprints. This matrix approach clarifies the steps and sustainability targets for the management of emission losses in buildings by presenting different parameters together, according to the type of building and possible implementation steps. Table 1 presents an example of a logical matrix for the proposed emissions inventory for buildings in the context of energy management and environmental sustainability.

### 2.2. Integrated Building Mechanical System Model

**Figure 2:**
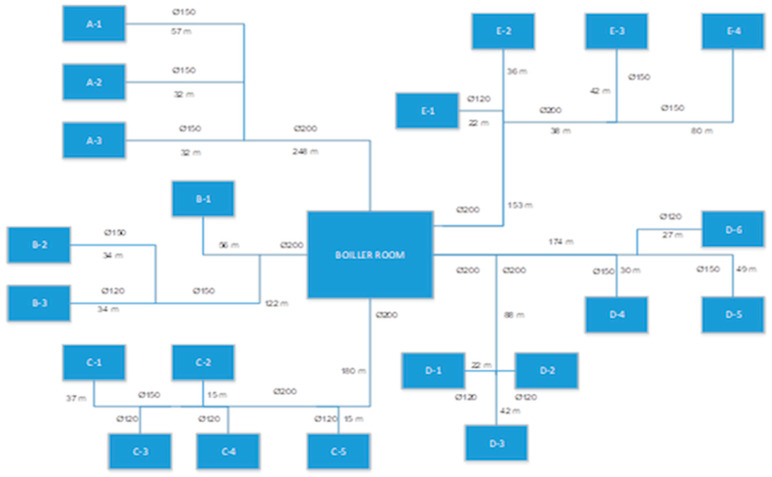
Flow chart of the main steam distribution line of premises with single center branch system [26].

The heat requirements in integrated buildings and the management elements of this energy are provided by the production of steam, boiling water, or hot water from a central boiler room. Energy-efficient applications in boiler systems include fluidized bed technologies for solid fuels and commensurately high-output burners for gas liquid or liquid fuel systems. Today, condensing technologies, especially for natural gas systems, as well as previously mixed combustion technologies, have contributed to the expected savings in energy consumption. The heat energy requirements of buildings are defined as water heating or hot water. Temperature limits for these requirements should be between 65 °C and 55 °C for heating and between 60 °C and 45 °C for hot water. The choice of steam should be limited for cooking and some requirement systems. This makes direct system selection for steam systems controversial. In this regard, Figure 2 below contains the system graph in which the losses caused by condensation in the outlines of the steam distribution system are examined, taking into account efficiency and energy costs.

The heat requirement of the plant is provided by the main distribution line with a length of 1898 m from a central boiler system. The system produces steam at an average of 155 °C; the system pressure is 3.1 Atm for 145 °C and 5 bar Atm for 165 °C. Distribution channels between buildings are laid in a closed structure, on average 2 m underground. The pipe parameters of the distribution lines in the system vary between 120 mm and 200 mm. The main distribution pipes in the duct lines are protected by glass wool insulation and waterproof exterior paint. The pipes are then connected to the buildings through conduits.

### 2.3. Assumptions and Reference Environment

To ensure the thermodynamic consistency and reproducibility of the proposed entropy-based assessment, the present study is conducted under a clearly defined set of assumptions and boundary conditions. These assumptions are introduced to isolate the dominant sources of irreversibility in the energy distribution system and to enable a transparent interpretation of entropy generation and exergy destruction within the integrated building framework.

#### 2.3.1. Steady-State Assumption

The analysis is performed under steady-state operating conditions for the main steam distribution network. It is assumed that mass flow rates, pressure levels, and temperature distributions remain constant over the considered operating period. This assumption is justified by the long-term continuous operation of central heating systems in integrated buildings, where transient effects are negligible compared to cumulative thermodynamic losses along distribution lines. Adopting steady-state conditions allows entropy generation and exergy destruction to be evaluated as representative indicators of average system performance.

#### 2.3.2. Reference Environment (Dead-State Conditions)

All exergy analyses and entropy generations are referred to as a defined ambient environment, commonly referred to as the dead state. The reference temperature and pressure are selected as T_0_ = 298 K and P_0_ = 1 atm, representing standard atmospheric conditions. These values are widely accepted in exergy-based analyses of energy building systems and provide a consistent basis for evaluating thermodynamic irreversibility and environmental interaction. The choice of reference environment ensures that calculated exergy destruction and entropy generation reflect the deviation of the system from equilibrium with its surroundings.

#### 2.3.3. Neglect of Chemical Exergy

In the present study, chemical exergy is neglected, and the analysis is limited to physical exergy components. This assumption is justified by the absence of chemical reactions within the steam distribution and condensate return lines. Since the working fluid (water/steam) undergoes only phase change and sensible heat transfer without any change in chemical composition, chemical exergy does not contribute to system irreversibility. Consequently, physical exergy associated with temperature and pressure differences is sufficient to characterize the dominant sources of inefficiency and entropy production in the distribution network.

#### 2.3.4. Condensate Formation and Heat Loss Model

Condensation formation in steam distribution lines occurs in two ways. Firstly, it occurs on the flow lines as a result of the heat transfer effect associated with viscous flows. Secondly, it occurs directly as a cyclical need of the system, by condensing the steam and sending it to the boiler via condensate pumps. In process analyses, thermal losses are a situation arising from heat transfer through insulated underground pipes with constant radial heat conduction and external convection effects. Therefore, condensation formation occurs as a direct result of irreversible heat transfer over finite temperature differences. This situation allows for the correlation of condensation load, which directly constitutes exergy destruction and depends on environmental conditions, with exergy destruction in exergy analyses.

## 3. Theoretical Analyses

Energy transformation in integrated building systems is inherently governed by thermodynamic processes in which energy quality degradation and irreversibility play a decisive role. In building-scale mechanical energy systems, thermal energy is transferred from a centralized heat source to end users through distribution networks, where unavoidable losses occur due to heat transfer, fluid friction, and phase change phenomena. While the first law of thermodynamics provides a quantitative description of energy conservation, the second law introduces entropy as a fundamental measure of irreversibility and degradation of energy quality. For this reason, the present analysis is formulated within an exergy, entropy framework, allowing the assessment of both system efficiency and environmental performance.

The overall exergy balance for the main steam distribution line of an integrated building system can be expressed as the sum of physical, chemical, kinetic, and potential exergy components of the flowing medium [27]:

(1) $${\dot{E}}_{x,\mathrm{total}}={\dot{E}}_{x,\mathrm{ph}}+{\dot{E}}_{x,\mathrm{ch}}+{\dot{E}}_{x,\mathrm{kin}}+{\dot{E}}_{x,\mathrm{pot}}$$

In the present study, exergy evaluation is conducted relative to a defined reference environment (dead state), and the thermodynamic state of the flow is characterized by its deviation from equilibrium with the surroundings [28].

(2) $$\psi =(h-h_{0})-T_{0}(s-s_{0})$$

Since no chemical reactions occur within the steam distribution and condensate return lines, chemical exergy is neglected, and the analysis focuses on physical exergy associated with temperature and pressure differences.

In steam-based distribution systems, energy loss is greatly affected by condensation formation along pipelines. Condensation occurs as a result of irreversible heat losses to the surrounding environment and represents a manifestation of irreversibility. Accordingly, the total thermal load due to condensation in the distribution line is defined as the sum of transient heat losses and condensation losses caused by flow-dependent viscous effects:

(3) $${\dot{Q}}_{\mathrm{total},\mathrm{cond}}=\sum {\dot{Q}}_{\mathrm{heat}}+\sum {\dot{Q}}_{\mathrm{steady}}$$

Here, ${\dot{Q}}_{\mathrm{heat}}$ represents the enthalpy load associated with the heating phase of the distribution components, while ${\dot{Q}}_{\mathrm{steady}}$ corresponds to condensation occurring under steady operating conditions. The transient condensation-related heat load is calculated as:

(4) $${\dot{Q}}_{\mathrm{heat}}=\frac{W(T_{a}-T_{\mathrm{out}})C_{p,\mathrm{pipe}}\;60}{h_{fg}\;\Delta t}$$

where W denotes the total mass of pipes, valves, and fittings; $T_{a}$ and $T_{\mathrm{out}}$ are the steam and ambient temperatures, respectively; $C_{p,\mathrm{pipe}}$ is the specific heat capacity of the pipe material; and Δt is the heating duration. Under steady-state conditions, condensation heat loss along insulated distribution lines is expressed as [29]:

(5) $${\dot{Q}}_{\mathrm{steady}}=\frac{\alpha I\;3.6}{4h_{fg}}$$

where Q_steady_ is the total heat load due to the enthalpy of vaporization, α is the heat loss coefficient dependent on pipe diameter, and I is the pipe length. Here, h_fg_ is the defined enthalpy of vaporization for the fluid. The thermal energy demand associated with condensation losses is supplied by fossil fuel combustion in central boiler systems. The corresponding annual fuel requirement is determined as:

(6) $$M_{y}=\frac{{\dot{Q}}_{\mathrm{year}}}{H_{u}}$$

where $H_{u}$ denotes the lower heating value of the fuel [30]. Fuel consumption directly determines greenhouse gas emissions, which are commonly quantified using CO_2_-equivalent emission factors recommended by the IPCC [31]. In building-scale assessments, specific emission generation (SEG) is employed as a normalized emission indicator, with natural gas typically characterized by an emission factor of 0.234 kgCO_2_eq/kWh [32]. The thermodynamic behavior of the system is further described using the energy balance derived from the first law of thermodynamics [33,34]:

(7) $$\dot{Q}-\dot{W}+\sum {\dot{E}}_{\mathrm{in}}-\sum {\dot{E}}_{\mathrm{out}}=0$$

When environmental interaction is considered, this balance is extended through exergy analysis, yielding the general exergy balance equation:

(8) $$\sum \left(1-\frac{T_{0}}{T_{k}}\right){\dot{Q}}_{k}-\dot{W}+\sum {\dot{E}}_{x,\mathrm{in}}-\sum {\dot{E}}_{x,\mathrm{out}}-{\dot{E}}_{x,\mathrm{dest}}=0$$

where ƩE_x,in_ refers to total exergy input based on total mass flow rate, ƩE_x,*out*_ refers to total exergy output of total mass flow rate and ƩEx_dest_ represents exergy destruction due to irreversibility of mass flow rate. According to the Gouy–Stodola theorem, exergy destruction is directly proportional to entropy generation that is given in the equation below [17,34]:

(9) $${\dot{E}}_{x,\mathrm{dest}}=T_{0}{\dot{S}}_{\mathrm{gen}}$$

This relationship forms the theoretical foundation of the present study, in which entropy generation is interpreted not only as a thermodynamic loss but also as a proxy for environmental burden.

Exergy efficiency is used to quantify the degree of irreversibility within the system and is defined as Equation (10) [35]:

(10) $$\eta_{Ex}=\frac{{\dot{E}}_{x,\mathrm{out}}}{{\dot{E}}_{x,\mathrm{in}}}=1-\frac{{\dot{E}}_{x,\mathrm{dest}}}{{\dot{E}}_{x,\mathrm{in}}}$$

It is an expected possible improvement target for system assessments in energy management. In this context, exergy efficiency defines potential depending on the maximum work obtained in relation to the environment in which the system is located. For this purpose, it defines the improvement potential (IP) defined as a target in the system for reducing irreversibility for the processes in Equation (11) brought to the literature by VanGool [36]:

(11) $$\dot{IP}=(1-\eta_{Ex})\left(\sum {\dot{E}}_{x,\mathrm{in}}-\sum {\dot{E}}_{x,\mathrm{out}}\right)$$

### Environmental Indicators for Sustainability

In fossil-fuel-based thermal systems, environmental impact is associated with exergy extinction in exergy analyses and is directly linked to irreversibility as an equation associated with entropy generated in dead-state conditions for environmental conditions. In fact, exergy destruction is an undefined consumption under normal conditions. However, it can be improved with advanced exergy analysis. It is envisaged that energy management can define it with a simpler criterion in order to use exergy analysis. Heat-generating processes contribute to the generation of entropy due to exergy destruction through their viscous flow properties on heat transfer and finite temperature differences. This leads to the generation of environmental thermal pollution due to the potential for exergy destruction from the system to the environment in thermodynamic processes. Based on this principle, Sogut [22] proposed that pollution should be interpreted as the potential for entropy generation of the system and as the ratio of the structure formulated by exergy destruction to the total exergy input under dead-state temperature. Following this approach, shortly defined as exergy destruction over the exergy input, the environmental performance index (EPI) is defined as:

(12) $$EPI=\frac{\sum {\dot{S}}_{\mathrm{gen}}}{\sum {\dot{E}}_{x,\mathrm{in}}}\;T_{0}$$

EPI is the ratio of entropy from exergy destruction to exergy input in the developed approach. This value defines the potential of thermal losses from the system to the environment in the range of 0–1. Here, the possible improvement target of this potential, which should be considered as a goal of energy management, is limited by the recyclability limit. Therefore, the possible entropy generation for reversibility conditions is obtained by calculating the exergy breakdown due to the reversibility condition. The potential between these two values due to the condition of reversibility and irreversibility has also been evaluated as heat-induced environmental pollution of the process and has been proposed as a traceable potential for energy management. In this context, in order to create a comparison based on the ratio of the total input exergy value of reversible conditions, the sustainability index (SI), which represents the entropy associated with idealized return conditions as a kind of exergy extinction, is introduced:

(13) $$SI=\frac{\sum {\dot{S}}_{\mathrm{gen},c}}{\sum {\dot{E}}_{x,\mathrm{in}}}\;T_{0}$$

There are many approaches to developing the optimization goal in the system for engineering goals. The target efficiency potential to be developed with the production of entropy, which is a form of exergy destruction in the thermodynamic sense, the system improvement associated with the IP value given in Equation (11), can then be made more limited between reversibility and irreversibility. In this context, the improvement potential has been modified and limited [36]:

(14) $$IP=(\eta_{\mathrm{Carnot}}-\eta_{II})\left({\dot{E}}_{x,\mathrm{in}}-{\dot{E}}_{x,\mathrm{out}}\right)$$

Finally, the energy efficiency ratio (θ), developed by Sogut [22], integrates entropy-based environmental indicators with improvement potential to assess energy management performance:

(15) $$\theta =\frac{\sum (EPI-SI)\cdot IP}{\sum {\dot{E}}_{x,\mathrm{in}}}$$

The energy efficiency ratio provides an entropy-based performance metric that enables simultaneous evaluation of energy efficiency improvement and environmental impact reduction, supporting sustainable energy management strategies in integrated building systems.

## 4. Results and Discussion

In integrated building systems, heating networks are interconnected through mechanical system rooms according to spatial and operational demand profiles. Thermal energy transport is predominantly achieved through steam-based distribution systems supplied by a central boiler plant. However, energy losses, particularly those occurring along steam distribution and condensate return lines, represent a significant inefficiency source. In this study, these losses are evaluated using an entropy-based thermodynamic framework, enabling the assessment of both energetic and environmental consequences.

**Figure 3:**
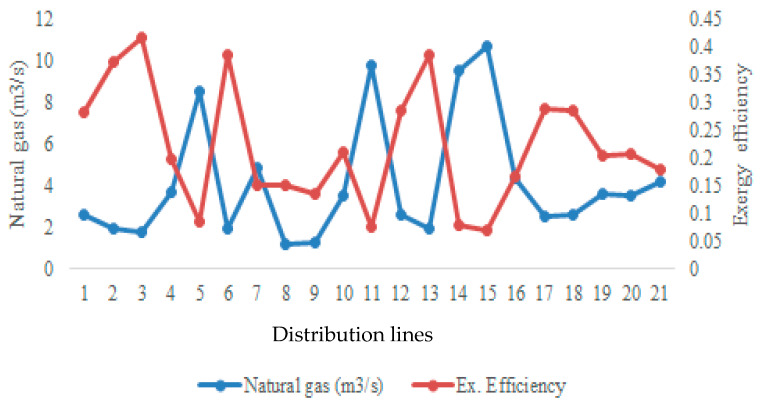
Exergy efficiency distributions depending on the consumption of distribution lines.

The annual natural gas consumption of the integrated building referenced in this study shows that the average natural gas consumption is 4.12 m^3^/h, steam flow values are 155 °C, condensate value is 92 °C, the ambient temperature is 10 °C, and the total steam and condensate distribution is approximately 1900 m, and it varies according to different pipe diameters and flow conditions. For the referenced model, an average condensate mass flow rate is taken as 6480.59 kg/s. For the building distribution for which the operating demand load was defined, an average value of 74 °C/50 °C was taken for the hot water system and the load value was analyzed with different temperature distributions in each distribution. Here, it was evaluated that each consumer had a different load and a distribution between 93 °C and 58 °C was modeled in the demand distribution. Here, what is expected from energy management is to determine the mass, temperature and load values for each user. It was evaluated that the system had steady state conditions and the boiler circuit was transformed from a steam structure to a 90 °C/70 °C system. All analyses based on these data proposed for the integrated structure were handled with derived data depending on these assumptions. The relationship between system demand and thermodynamic performance is illustrated by the distribution of exergy efficiency as a function of energy consumption (Figure 3). However, especially in exergy analyses, only physical exergy loads were taken into account and the condensate load difference was processed. In the study, especially the insulation of the distribution lines and possible technical problems were neglected in the analysis. In the analyses, each line was modeled as a user and the load values were analyzed accordingly.

### 4.1. Exergy Efficiency and Irreversibility Characteristics

As shown in Figure 3, the average exergy efficiency of the distribution system is 21.99%, indicating that a substantial portion of the supplied exergy is destroyed due to irreversibility. Although exergy efficiency increases with decreasing exergy load, the overall system performance remains dominated by high inefficiency under typical operating conditions. This observation highlights the necessity of evaluating exergy destruction alongside efficiency metrics.

**Figure 4:**
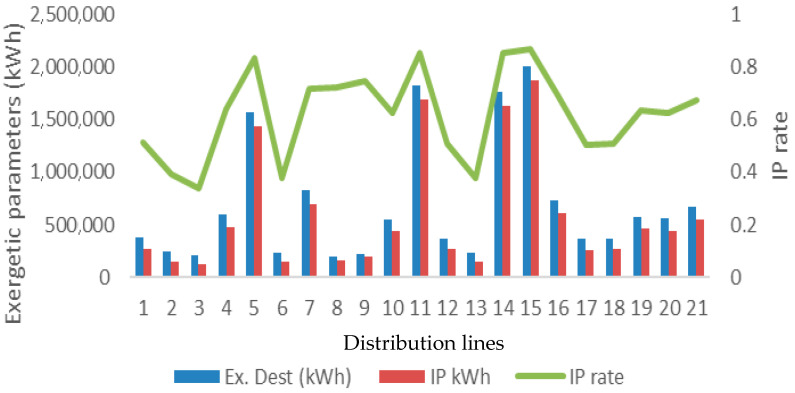
Exergy destruction together with IP distribution.

The corresponding distributions of exergy destruction and improvement potential (IP) are presented in Figure 4. The results reveal an average exergy destruction rate of 78.01%, confirming the dominance of irreversible processes within the distribution network. When combined with IP analysis, this irreversibility translates into an improvement potential of 62.03% attributable to exergy destruction and 50.80% associated with total exergy flow. These values indicate a substantial margin for thermodynamic and operational improvement, particularly in relation to entropy generation mechanisms.

### 4.2. Entropy Generation and Environmental Interpretation

**Figure 5:**
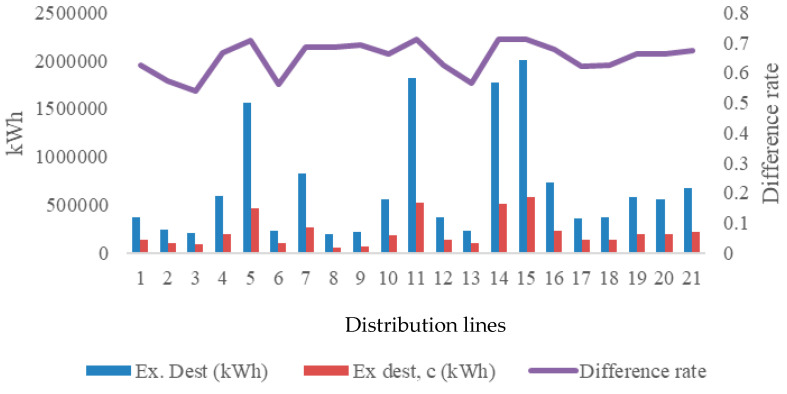
Exergy destruction impact for distribution lines.

**Figure 6:**
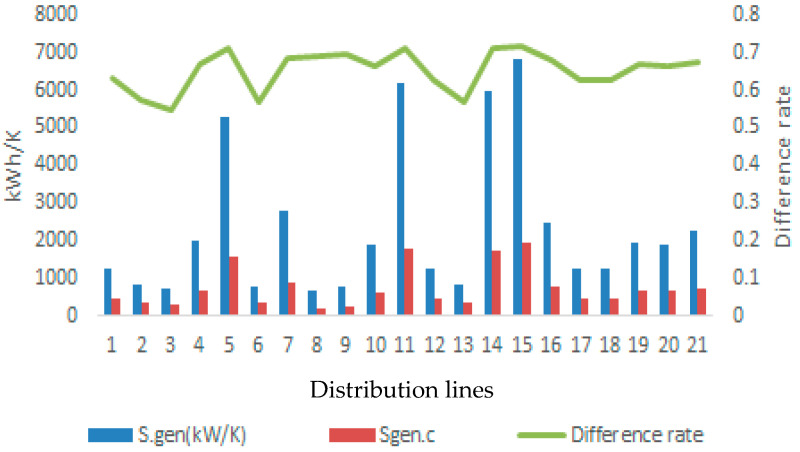
Entropy generation of distribution lines for irreversible and reversible processes.

Figure 5 shows the distribution of exergy extinction between reversible and irreversible operating conditions, giving the average irreversibility effect associated with condensation formation along the distribution lines of 64.92%. This potential is the average entropy generation potential for the two limit values of the direct condensate charge. This effect is a result of exergy destruction caused by irreversible heat transfer and phase change processes. Of course, this value indicates the entropy production potential in the environmental conditions found for distribution lines. The rate of entropy production under reversible and irreversible conditions is shown in Figure 6. Here, the average rate of entropy change is calculated as 65.28%. This value constitutes a critical indicator of environmental impact, as entropy generation is treated in this study as a thermodynamic proxy for pollution. Under real operating conditions, irreversible entropy production reflects additional fuel consumption and increased emissions, particularly when condensate-related thermal losses are considered.

### 4.3. Sensitivity of Entropy Load to Condensate Rate

To evaluate the robustness and generality of the proposed entropy-based framework, a sensitivity analysis is conducted with respect to key governing parameters.

A ±10% variation in condensate mass flow rate results in a proportional change in entropy generation, leading to an approximate ±6–8% variation in total entropy load. This sensitivity confirms that condensate formation is a dominant contributor to entropy production and environmental impact within steam distribution systems.

Similarly, a ±5 K change in the reference environment temperature (T_0_) induces a measurable variation in the environmental performance index (EPI). An increase in T_0_ leads to higher calculated entropy-related environmental impact, whereas a decrease in T_0_ reduces EPI values. This outcome highlights the importance of reference environment selection and confirms the thermodynamic consistency of the entropy-based indices. These sensitivity trends demonstrate that, while numerical values vary with operating and reference conditions, the qualitative conclusions regarding irreversibility dominance and environmental burden remain unchanged.

**Figure 7:**
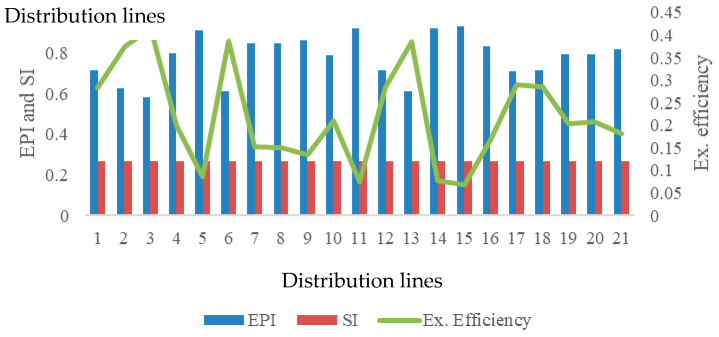
Environmental impact assessment based on exergy efficiency of distribution lines.

The average rate of change was found to be 65.28% depending on reversible and irreversible conditions, which is an important criterion in terms of environmental impact. The irreversible conditions of entropy are also realized as a potential of pollution produced for real processes. Especially when the demand loads of the buildings are taken into account, condensate processes also show an effect of fuel consumption as thermal pollution. Here, pollution in terms of thermodynamic effect indicates a potential of fuel-induced environmental pollution directly related to the condensate load of the structure. This value was evaluated with two indices developed in particular. In these indices, EPI directly indicates the potential of pollution under real conditions, while the SI value indicates the value that should be reached due to reversibility and that the pollution has reached the dimensions to be eliminated. In this context, Figure 7 points to a framework in which these two indices are evaluated together with exergy efficiency.

### 4.4. Environmental Performance Indices and Energy Management Implications

Environmental impact assessment based on exergy efficiency and entropy-derived indices is presented in Figure 7. A clear inverse relationship between EPI and exergy efficiency is observed. While the average SI value is 0.268, the corresponding EPI value reaches 0.835, indicating a high pollution potential under real operating conditions. Even under minimum entropy production scenarios, EPI remains at 0.583, exceeding the sustainability threshold by more than 120% relative to SI.

**Figure 8:**
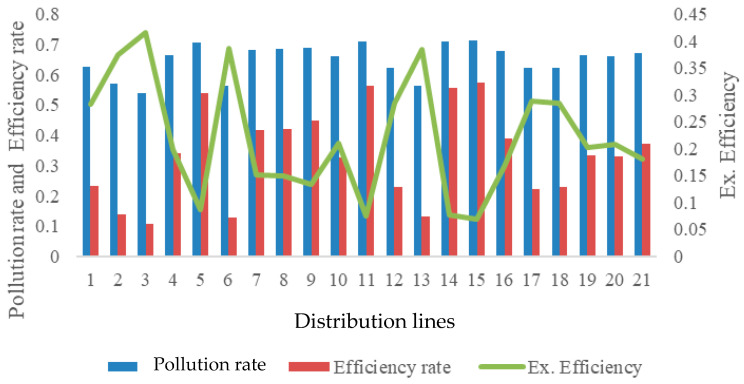
Pollution rate and efficiency rate for energy management.

The highest pollution potential, reaching 41.70%, corresponds to the lowest exergy efficiency levels, demonstrating that improvements in thermodynamic efficiency directly contribute to reduced environmental impact. These findings confirm that energy efficiency metrics alone are insufficient to characterize environmental performance unless irreversibility and entropy generation are explicitly considered. Pollution rate and efficiency rate for energy management is shown in Figure 8.

Based on entropy-based assessment, the average rate of contamination associated with condensate-induced entropy production alone is based on the difference in entropy production between the reversible and irreversibility processes, with the mean potential calculated to be 64.93%. On the other hand, the target energy efficiency level for effective energy management has been determined as 33.48% on average according to Equation (15). These values provide actionable indicators for operational decision making and performance monitoring in integrated building systems.

### 4.5. Implications for Entropy Theory and Sustainability

One of the important contributions of this study is the interpretation of the entropy generation that occurs with exergy extinction as an environmental measure rather than a purely thermodynamic abstraction. While traditional energy analysis quantifies energy amounts, it does not distinguish between usable and lost potential. On the other hand, the production of entropy due to environmental conditions as a result of exergy destruction defines exergy destruction and this loss irreversibility can be directly related to environmental conditions. This study shows that irreversibility can be systematically interpreted as pollution between recyclability and non-recyclability because entropy production redirects additional fuel consumption, increases emissions, and disrupts environmental interaction. By introducing entropy-based environmental performance indices, the proposed framework defines the quantitative potential between thermodynamic theory and sustainability assessment.

From the perspective of entropy theory, these findings reinforce the concept that minimizing entropy production is not just an efficiency goal but also an environmental imperative. The distinction between energy-based and entropy-based assessment becomes particularly evident in building-scale systems, where large energy flows can coexist with disproportionately high environmental impacts due to their return. In this context, the entropy approach broadens its applicability by demonstrating its practical importance in environmental performance assessment and energy management of integrated buildings. The proposed approach offers a concrete point of interpretation for entropy generation, enabling its integration into carbon purification strategies and sustainable building operation. In this context, a comparison table has been added for the sustainability assessments of energy management. Energy management mostly prefers regression analysis to reveal efficiency performance. However, entropy production reveals more solid data for process analysis. In this context, a comparative evaluation table for energy management, Table 2, is created and presented.

## 5. Conclusions

Integrated buildings exhibit high energy density due to their multifunctional operation, making energy distribution efficiency a critical determinant of both system performance and environmental impact. In steam-based heating networks, energy degradation associated with condensate formation along distribution lines represents a dominant source of irreversibility. This study addressed this challenge by applying an entropy-based exergy framework to explicitly quantify the environmental impact and energy efficiency potential arising from condensate-induced irreversibility in integrated building systems.

The results show that the average exergy efficiency of the researched distribution network is limited to 21.99%, while the exergy extinction reaches 78.01%, indicating a high exergy destruction for the distribution lines. The improvement potential developed as a target shows a value of 62.03% according to exergy extinction and 50.80% according to exergy flow. A comprehensive assessment should be needed in conjunction with operational processes for possible opportunities for energy management. The analyses also show that the rate of entropy production, a form of condensate-associated exergy destruction, reaches 65.28%.

From an environmental point of view, the pollution potential based on exergy destruction, which is shaped by the production of entropy associated with condensation formation, shows a potential of 64.93%, while the target energy efficiency level that can be developed by energy management in the building with effective measures is defined as 33.48%. These findings are presented only as an alternative to traditional energy efficiency measures in defining the true environmental burden of building energy systems. This is also suggested as a criterion to be evaluated in terms of entropy production as a form of exergy destruction.

Beyond the quantitative results, the main contribution of this study is in the conceptual interpretation of the entropy value produced by the potential defined by exergy destruction as an environmental burden. By establishing a direct link between irreversibility, condensation-induced exergy degradation and entropy production and pollution potential, the proposed framework for energy management is that the value of entropy production obtained by exergy analyses is defined by an operational indicator for sustainability assessments. The developed indices associated with exergy destruction and associated with entropy production (EPI and SI) are evaluated to provide a systematic and physically based method for translating thermodynamic losses into interpretable environmental performance metrics.

Overall, this study is a modeling exercise in which energy management will show that minimizing entropy production is not only a goal of thermodynamic efficiency but can also be used as a benchmark for environmental sustainability in integrated buildings, as can be seen in Table 2. The proposed approach aimed to enhance the applicability of entropy generation in building-scale energy management and develop a fuel-focused benchmark for decarbonization-oriented decision making. Future research is to develop entropy generation in conjunction with exergo-economic life cycle analyses and strengthen its role in sustainable energy system design.

## Figures and Tables

**Table 1:** Example of emission inventory logical matrix for buildings.

Processes	Source Structure	Emission Type	Source Type	Monitoring Methods	Sustainability Goals
Heating and Cooling Systems	Natural gas, oil and other fossil fuels	Carbon dioxide (CO_2_)	Fossil fuels	Measurements, gas meters, heating systems data	Reduce carbon emissions by 55% (2030 target)
Electricity Consumption	Electrical appliances, lighting, HVAC, appliances	Carbon dioxide (CO_2_), nitrogen oxide (NOx), sulfur dioxide (SO_2_)	Electricity usage	Consumption data, system analysis	Increasing the proportion of renewable energy (2030: 40% target)
Building Insulation and Structural Systems	Inadequate insulation, windows, doors	Carbon dioxide (CO_2_)	Energy-inefficient structures	Energy loss analysis, Thermal monitoring	Increase build envelope efficiency by 30% (2027 target)
Water Heating Systems	Electric water heaters, gas water heaters	Carbon dioxide (CO_2_)	Electricity, gas	Consumption monitoring	Reducing water consumption by 25% (2030 target)
Need for Cooling	Cooling systems (air conditioner, chiller, etc.)	Carbon dioxide (CO_2_)	Electricity	HVAC system data, Energy measurements	Reduce cooling energy consumption by 20% (2027 target)
Materials and Construction Processes	Production of construction materials (cement, steel, etc.)	Carbon dioxide (CO_2_), methane (CH_4_)	Production of construction materials	Material emission supply chain data	Increase the use of zero-carbon construction materials by 50% (2030 target)
Waste Management and Recycling	Building waste, recycling processes	Methane (CH_4_), carbon dioxide (CO_2_)	Waste management and recycling	Waste types and amounts, recycling rate	Increase recycling rate by 75% (2027 target)
Renewable Energy Systems	Solar, wind, geothermal, etc.	Carbon dioxide (CO_2_) (for negative emissions)	Renewable energy generation	Renewable energy production data, system efficiency	Increase renewable energy capacity by 40% (2040 target)

**Table 2:** Comparative evaluation table for energy analysis for energy management.

Feature	Classical Energy Audit (Regression Analysis)	Entropy-Generation-Based Approach
Main Purpose	To model the linear relationship between energy consumption and independent variables. (The independent variable can be non-energy data.)	To analyze exergy consumption, inefficiencies and environmental impacts for processes directly linked to exergy destruction by entropy generation.
Data Type Used	Statistical relationship of energy consumption with variable factors or other independent variables.	Quantitative dimension of entropy production based on losses in energy conversions and consumption and their exergy destruction.
Analysis Approach	Defines the statistical relationship, measures the effect of independent variables on the dependent variable in energy consumption.	Handles energy consumption with thermodynamic analysis. Quantitatively illustrates the irreversibility and environmental impacts of energy conversions.
Environmental Impacts	Environmental impacts are often shaped by a fuel emission multiplier, without considering efficiency.	The production of entropy is directly associated with environmental influences. Quantitative potential was developed with a pollution factor and indicators to monitor the potential with EPI and SI. The connection of exergy destruction between emissions has been developed as a quantitative value.
Efficiency Measurement	Energy efficiency is statistical potential with a generalization based on the reduction of energy consumption.	Efficiency is expressed by exergy analyses, the efficiency ratio is brought to a correlation between the entropy ratio and the improvement potential.
Environmental Sustainability	Environmental impacts are often addressed indirectly to fuel consumption, not directly addressing efficiency.	Entropy production is directly related to exergy destruction and environmental effects, producing a quantitative value between exergy consumption and emissions.
Modeling and Application Area	With statistical modeling, general trends are determined and linear relationships are revealed. However, it is independent of processes.	With thermodynamic modeling, energy flows of fuels and entropy production are calculated by exergy analysis, providing more concrete quantitative data on the environmental performance of buildings.
Management Applicability	It is used in the general assessment of energy consumption, it is a reference for policies to reduce energy consumption.	More concrete strategies can be developed for environmental sustainability, supporting practices that can reduce energy efficiency and environmental impact by reducing exergy destruction, reducing entropy production.
Implementation Challenges	A large number of recorded data are needed to understand the linear relationship between independent variables and dependent variables. Field work is required for correlation.	Entropy calculations for exergy analyses and the development of energy conversions require real data and detailed analyses. Detailed engineering analyses for entropy generation require more complex and comprehensive modeling.

## Data Availability

The original contributions presented in this study are included in the article. Further inquiries can be directed to the corresponding author.

## References

[1] Lin S., Zhang Y., Chen X., Pan C., Dong X., Xie X., Chen L. (2025). Review and decision-making tree for methods to balance indoor environmental comfort and energy conservation during building operation. Sustainability.

[2] Bandaranayake S., Navaratnam S., Munmulla T., Zhang G., Aye L. (2025). Innovative technologies for building envelope to enhance the thermal performance of a modular house in Australia. Energies.

[3] Gliss C., Bachmann C., Ciattaglia S., Drumm B., Camacho Gracia M., Moscato I., Mull T., Palermo I. (2022). Integrated design of tokamak building concepts including ex-vessel maintenance. Fusion Eng. Des..

[4] Gerigk M. (2017). Multi-criteria approach in multifunctional building design process. IOP Conf. Ser. Mater. Sci. Eng..

[5] Li J. (2024). Optimization strategy of property energy management based on artificial intelligence. Energy Inform..

[6] Khan O., Parvez M., Alansari M., Farid M., Devarajan Y., Thanappan S. (2023). Application of artificial intelligence in green building concept for energy auditing using drone technology under different environmental conditions. Sci. Rep..

[7] Bazazzadeh H., Nadolny A., Safaei S.S.H. (2021). Climate change and building energy consumption: A review of the impact of weather parameters influenced by climate change on household heating and cooling demands of buildings. Eur. J. Sustain. Dev..

[8] Vatandaş S., Sogut M.Z., Onen Y.E., Sogut M.Z. (2025). The role of energy audits in energy management studies towards decarbonisation. The Role of Exergy and Energy in Sustainability.

[9] De la Cruz I., Ugalde-Loo C.E. (2021). District heating and cooling systems. Microgrids and Local Energy Systems.

[10] Horry R., Booth C.A., Mahamadu A., Olomolaiye P. (2022). Environmental management systems in the architectural, engineering and construction sectors: A roadmap to aid the delivery of the sustainable development goals. Environ. Dev. Sustain..

[11] Kas Ö., Sogut M.Z., Sogut M.Z., Koray M. (2025). Assessment of thermo-economic performance with sustainable heat management in trigeneration processes. Energy Rationality and Management for Decarbonization.

[12] Prozuments A., Borodinecs A., Zaharovs S., Banionis K., Monstvilas E., Norvaišienė R. (2023). Evaluating reduction in thermal energy consumption across renovated buildings in Latvia and Lithuania. Buildings.

[13] Sogut M.Z., Sogut M.Z., Koray M. (2025). Walk-through audit model for enterprise’s decision processes regarding energy management. Energy Rationality and Management for Decarbonization.

[14] Umar D.A., Abubakar M.M. (2014). Effects of energy utilization on the environment. Discovery.

[15] Subbotina D.S., Subbotin A.S. (2018). Features of formation and functioning of the integrated structures in construction. IOP Conf. Ser. Mater. Sci. Eng..

[16] Rehman U.U., Faria P., Gomes L., Vale Z. (2025). Future of energy management models in smart homes: A systematic literature review of research trends, gaps, and future directions. Process Integr. Optim. Sustain..

[17] Rosen M.A., Dincer I. (2001). Exergy as the confluence of energy, environment and sustainable development. Exergy.

[18] Cornelissen R.L. (1997). Thermodynamics and Sustainable Development. Ph.D. Thesis.

[19] Bejan A. (2013). Entropy Generation Minimization: The Method of Thermodynamic Optimization of Finite-Size Systems and Finite-Time Processes.

[20] Sciubba E. (2011). Entropy Generation Minimization as a Design Tool. Part 1: Analysis of Different Configurations of Branched and Non-branched Laminar Isothermal Flow Through a Circular Pipe. Int. J. Thermodyn..

[21] Feng H., Chen X., Heck P., Miao H. (2014). An Entropy-Perspective Study on the Sustainable Development Potential of Tourism Destination Ecosystem in Dunhuang, China. Sustainability.

[22] Sogut M.Z. (2021). New approach for assessment of environmental effects based on entropy optimization of jet engine. Energy.

[23] Yılmaz İ.C. (2019). Discovering hidden patterns in Turkish construction projects delays related to project characteristic. J. Sustain. Constr. Mater. Technol..

[24] European Environment Agency (EEA) (2024). Member States’ Greenhouse Gas (GHG) Emission Projections 2024.

[25] European Environment Agency (EEA) (2025). National Emissions Reported to the UNFCCC and to the EU Under the Governance Regulation; Version 3.0.

[26] Sogut M.Z., Ekmekci İ., Karakoç T.H. Integrated building entropy production considering main distrubition line with system preference. Proceedings of the 7th Global Conference on Global Warming (GCGW-2018).

[27] Balkan F., Colak N., Hepbasli A. (2005). Performance evaluation of a triple-effect evaporator with forward feed using exergy analysis. Int. J. Energy Res..

[28] Szargut J., Morris D.R., Steward F.R. (1988). Exergy Analysis of Thermal and Metallurgical Processes.

[29] Intervalf (2011). Steam Installation and Steam Equipment Handbook.

[30] Koçak S., Şaşmaz C., Atmaca İ. (2012). Technical and economic aspects of a shopping center insulated in accordance with TS 825 for different degree-day zones. Install. Eng. Mag..

[31] Özkan M. (2006). Greenhouse Gases Emission Inventory Working Group Draft Report.

[32] Bayram M. Reference building concept and energy classification in BEP-TR calculation method. Proceedings of the X. National Plumbing Engineering Congress.

[33] Çengel Y.A., Boles M.A. (2014). Thermodynamics: An Engineering Approach.

[34] Moran M.J., Shapiro H.N., Boettner D.D., Bailey M.B. (2011). Fundamentals of Engineering Thermodynamics.

[35] Dinçer İ., Rosen M.A. (2012). Exergy: Energy, Environment and Sustainable Development.

[36] Van Gool W. (1997). Energy policy: Fair allocation of resources and obligations. Energy Policy.

